# At the molecular resolution with MINFLUX?

**DOI:** 10.1098/rsta.2020.0145

**Published:** 2022-04-04

**Authors:** Kirti Prakash

**Affiliations:** ^1^ National Physical Laboratory, TW11 0LW Teddington, UK; ^2^ Department of Chemistry, University of Cambridge, CB2 1EW Cambridge, UK; ^3^ Joint Integrated Pathology Unit, Centre for Molecular Pathology, The Royal Marsden Trust and Institute of Cancer Research, Sutton SM2 5NG, UK

**Keywords:** MINFLUX, STED, SMLM, localization precision, image resolution, super-resolution imaging

## Abstract

MINFLUX is purported as the next revolutionary fluorescence microscopy technique claiming a spatial resolution in the range of 1–3 nm in fixed and living cells. Though the claim of molecular resolution is attractive, I am concerned whether true 1 nm resolution has been attained. Here, I compare the performance with other super-resolution methods focusing particularly on spatial resolution claims, subjective filtering of localizations, detection versus labelling efficiency and the possible limitations when imaging biological samples containing densely labelled structures. I hope the analysis and evaluation parameters presented here are not only useful for future research directions for single-molecule techniques but also microscope users, developers and core facility managers when deciding on an investment for the next ‘state-of-the-art’ instrument.

This article is part of the Theo Murphy meeting issue ‘Super-resolution structured illumination microscopy (part 2)’.

## 1. Introduction

The spatial resolution of light microscopy is continuously being pushed with the development of new technologies. The significant milestones have been confocal laser scanning microscopy [1], 2-photon microscopy [2], 4Pi microscopy [3], stimulated emission depletion (STED) microscopy [4], zero-point STED [5], structured illumination microscopy (SIM) [6,7] and single-molecule localization microscopy (SMLM) [8,9]. Recently, hybrid super-resolution microscopy techniques have further pushed the spatial resolution down to a few nanometers. These can be further divided into direct combinations of SMLM+STED (for scanning the position of the molecule with a donut) as in MINFLUX [10] and SMLM+SIM as in SIMFLUX [11,12] or sequential correlative combinations of SIM and SMLM [13] and STED and SMLM [14].

The major advantage of the direct combination is in resolution enhancement by increasing the information per photon. For readers interested in the technical details of these methods, following are some excellent recent reviews on this topic [15–19]. MINFLUX and SIMFLUX have demonstrated a resolution of less than 10 nm on synthetic structures like DNA origami and over 40 nm on biological structures. However, as most of the biologically relevant structures are novel and lie between 10 and 200 nm range, sequential correlative methods have a distinct advantage to independently validate new morphological findings using an orthogonal technique with a reasonably close resolution.

MINFLUX is presently the most photon efficient method to localize molecules and the aim of this article is not to argue otherwise. Here, I evaluate MINFLUX on the following four broad categories:

1. New biological insights through molecular resolution
2. *A priori* structural information and subjective event filtering
3. Multi-colour, three-dimensional, live imaging and the caveats when imaging ideal, well-defined structures
4. Spatial resolution versus localization precision, density

I hope this detailed categorization helps scientists to evaluate whether MINFLUX is the right microscopy technique for their research.

## 2. New biological insights through molecular resolution?

**Figure 1.**
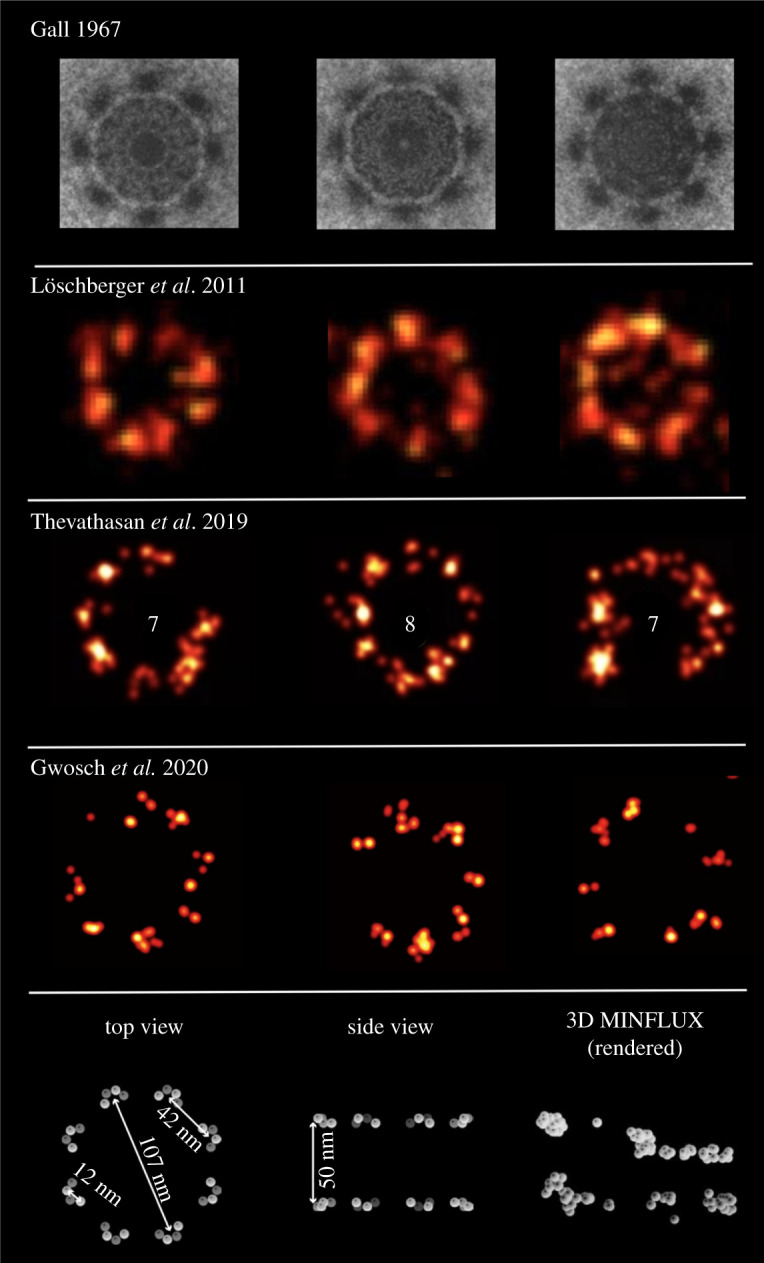
The need for molecular resolution? Nuclear pores across different imaging modalities: *(First row)* Nuclear envelope from amphibian oocyte imaged with electron microscopy. The eightfold symmetry of the nuclear pores is visible. The pore perimeter shows up as an octagon. Image adapted from Gall [21]. *(Second row)* Membrane protein gp210 from amphibian oocyte imaged with dSTORM (Alexa Fluor 647). The eightfold symmetry and the circular structures of NPCs is generally seen. The outer diameter is ∼120 nm and FWHM of gp210 is ∼30 nm. Image adapted from Löschberger *et al.* [22]. *(Third row)* Nup96 endogenously labelled with SNAP-tag (Alexa Fluor 647) in U2OS cell lines. Eight- and seven-component pores are more commonly observed. The effective labelling efficiencies for SNAP-Alexa Fluor 647 was ∼60%. Image adapted from Thevathasan *et al.* [23]. *(Fourth row)* MINFLUX imaging of U2OS cell expressing Nup96–SNAP labelled with Alexa Fluor 647. Six and seven component nuclear pores are more prominent, raising questions on the detection efficiency of the method. A special image rendering was used to visualize the four individual copies per subunit of NPCs, which appears as a blob for multi-colour MINFLUX imaging, see figure 2. Cell line and labelling strategy the same as in Thevathasan *et al.* [23]. Image adapted from Gwosch *et al.* [20]. *(Fifth row)* A schematic of NPCs with the dimensions Nup-96, taken from Thevathasan *et al.* [23]. A three-dimensional MINFLUX ‘rendered’ data presented for comparison from Gwosch *et al.* [20]. Colourmap removed for a fair comparison. Note the highly clustered, under-sampled and uneven distribution of well-defined periodic nuclear pores. (Online version in colour.)

The primary highlight of the paper by Gwosch *et al.* [20] is the ability to resolve the individual components of nuclear pore complexes (NPCs) at the molecular scale (figure 1, fourth row). NPCs have eight subunits, each with four copies of Nup96 and MINFLUX claims to have the resolution to resolve these four copies. Here, I re-examine this claim to probe if this molecular resolution provided new biological insights or was simply a visualization enhancement scheme.

### (a) New insights into nuclear pore biology

NPCs are symmetrical structures of eight subunits arranged in an octagonal geometry with an outer diameter of approximately 120 nm (see figure 1, fifth row for a detailed schematic). There are 32 copies of Nup96 per NPC with each of the eight subunits having four copies. These four copies are roughly around 12 nm in diameter. The organization of the four copies in the individual subunits is still unknown. With a 1–3 nm resolution range of MINFLUX, a general expectation is to gain structural insights into the organization of individual components of the NPCs. Do the four copies have some ordered organization (for example, a tetrahedron) or are they randomly distributed? As no observations on the internal organization of these copies (12 nm structures) are made, it raises questions on the actual usefulness of 1-nm resolution of MINFLUX to provide new structural insights.

### (b) Need for independent validation

It is worth noting that no independent validation of molecular copies or subunits of NPCs was done in Gwosch *et al.* [20] using either STED or SMLM. Moreover, Thevathasan *et al.* [23] used the same cell line (U2OS) and labelling strategy (Nup96-SNAP) as in Gwosch *et al.* [20] (same authors) and provide an easy basis for direct comparison. The molecular components are 12 nm in diameter and 42 nm apart, so should be easily resolved by both MINFLUX and SMLM.

As far as the organization of eight subunits of NPCs is concerned, they have been resolved with electron microscopy (EM) and single-molecule localization microscopy (SMLM) roughly 50 and 10 years ago, respectively [21,22]. For a comparison of NPCs using EM and SMLM see figure 1.

### (c) Detection versus labelling efficiency

Gwosch *et al.* [20] used Nup96 endogenously labelled with SNAP-tag (Alexa Fluor 647) in U2OS cell lines, the same as in Thevathasan *et al.* [23]. As a general observation, 2–3 components per pore are often missing in MINFLUX data (figure 1, fourth row). MINFLUX images show NPCs with six and seven components, which is not the case with SMLM data. This raises concerns on the detection efficiency of MINFLUX and the role of event filtering (see next section). If the missing components are due to labelling efficiency of SNAP-tag then this would be observed in the SMLM data of Thevathasan *et al.* [23]. Moreover, if the lack of labelling would be random, 1–2 copies of Nup96 would be missing per subunit of the pore, reducing the intensity but not the entire NPC subunit. It is very likely that the subjective filtering of localizations based on photon counts and the distance from the estimated position (structure assumption) leads to the missing pore subunit.

### (d) MINFLUX and the case of fewer photons

Gwosch *et al.* [20] state that MINFLUX requires fewer detected photons when compared to camera-based localization methods like PALM/STORM. So it is applicable to a large range of fluorophores and labelling strategies and is ‘bound to be a cornerstone, if not the vanguard, of nanometer-scale fluorescence’. However, as of now, MINFLUX has made use of one of the brightest and the best fluorophores for localization microscopy, namely Alexa Fluor 647, and has not done any systematic comparison between dyes or different super-resolution methods. In Balzarotti *et al.* [10] MINFLUX comparison with PALM/STORM (using DNA origami) was only a simulation and no experimental data was presented. It is worth noting that the authors as part of another paper [23] created four cell lines for Nup96 which also includes mEGFP. mEGFP and other organic dyes have poor emission stability and as MINFLUX localization precision lies on successive measurements of intensities over each ON cycle, it will be beneficial for the community to see the performance of these dyes. For a relative comparison of SMLM and MINFLUX using taxane analogues coupled to red fluorescent dyes (HMSiR) [24], please see figure 3.

**Figure 2.**
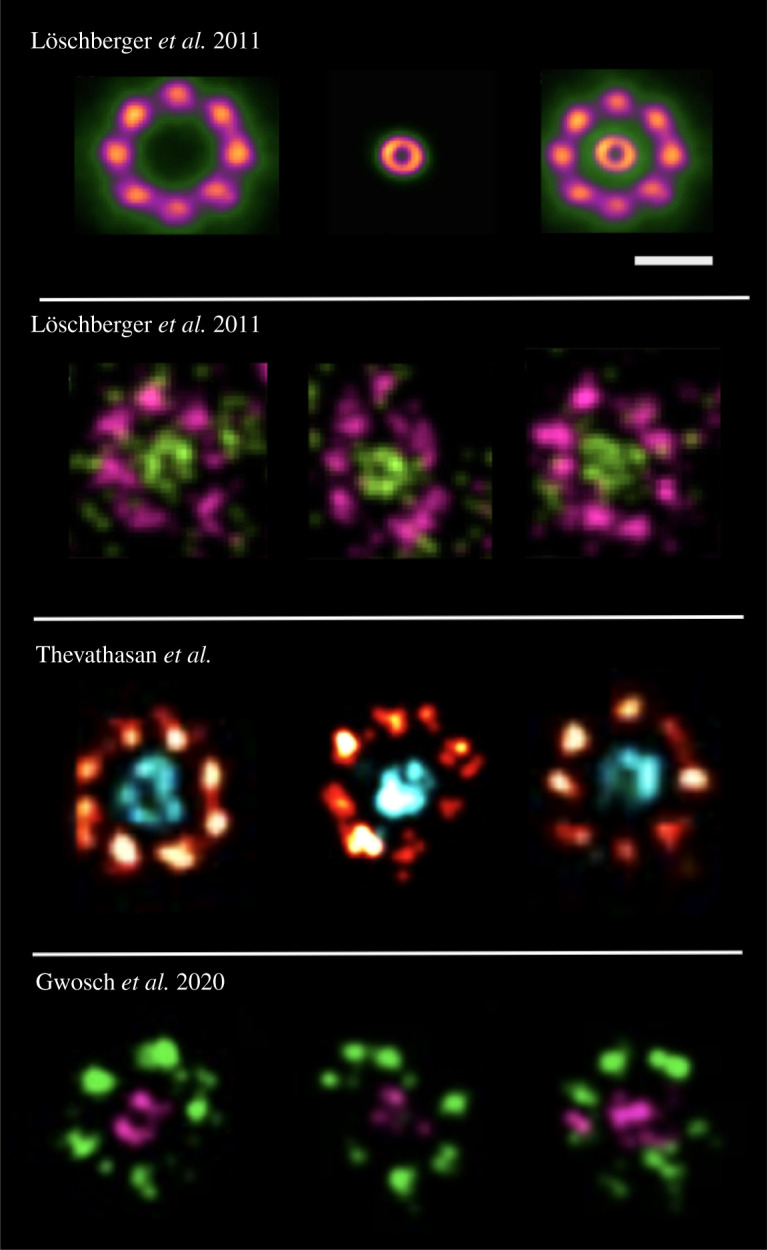
The missing molecular components with two-colour MINFLUX imaging: *(First row)* Schematic for gp210 (the outer ring) and WGA (the central channel) of the NPC. The outer ring (gp210) has an average diameter of ∼120 nm. The diameter of the inner ring (WGA) is 41±7 nm (true value ∼50 nm). Image adapted from Löschberger *et al.* [22]. Scale bar, 100 nm. *(Second row)* dSTORM images of WGA labelled with ATTO 520 (green) and gp210 labelled with Alexa Fluor 647 (pink) in amphibian oocytes. Both the outer ring and inner channel are visible [22]. *(Third row)* Two-colour SMLM image of Nup96-SNAP labelled with Alexa Fluor 647 (red) and WGA-CF680 (cyan) in U2OS cell lines. The outer ring is clearly visible and the inner ring is also visible in most cases. Image adapted from Thevathasan *et al.* [23]. *(Fourth row)* Two-colour MINFLUX imaging of U2OS cell expressing Nup96–SNAP labelled with Alexa Fluor 647 and WGA conjugated to CF680. The outer ring where each of the eight NPC subunits have four copies (as in figure 1) now appear as a blob. The inner ring (WGA) also aggregates as blobs. Image adapted from Gwosch *et al.* [20]. It must be noted that the inner and outer of nuclear pores in Gwosch *et al.* [20] were imaged three-dimensionally, while in Löschberger *et al.* [22] and Thevathasan *et al.* [23] they were in two dimensions. (Online version in colour.)

**Figure 3.**
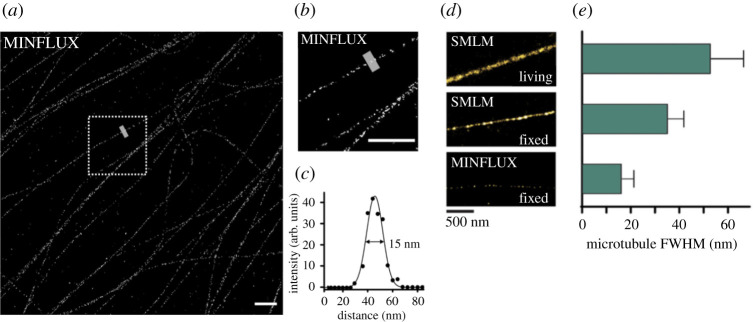
Localization precision versus localization density: (*a*,*b*) MINFLUX imaging of microtubules using HMSiR probe. (*c*) FWHM of inner diameter of microtubule. The standard estimate for inner diameter of microtubule is 17 nm (25 nm for the outer diameter diameter). (*d*) A comparison of SMLM and MINFLUX using the same HMSiR probe. (*e*) The corresponding FWHM of microtubules (live, fixed for SMLM and fixed for MINFLUX). Though MINFLUX achieves better precision than SMLM, the localization probability and density remain poor. One of the overlooked parameters in single-molecule imaging is sampling and the requirement to have a minimum number of localizations to decipher the underlying structure. Here, *a priori* knowledge of the hollow cylindrical structure of the microtubule is assumed to map the dots-on-line configuration to the under-sampled data. The successive measurement of intensities especially of dyes with poor emission stability is also likely to have an impact on the sampling and localization density. Figure was kindly provided by Gražvydas Lukinavičius and data from Gerasimaite *et al.* [24]. (Online version in colour.)

## 3. *A priori* structural information and event filtering

For years, in electron and single-molecule localization microscopy, filtering of imaging data has been done to enhance contrast and optimize visualization. In this section, I highlight how event filtering can be used to attain higher localization precision, purport new biological structures not present in the raw data and the need for blind samples to standardize the resolution claims.

### (a) Localization filtering at manually defined positions

**Figure 4.**
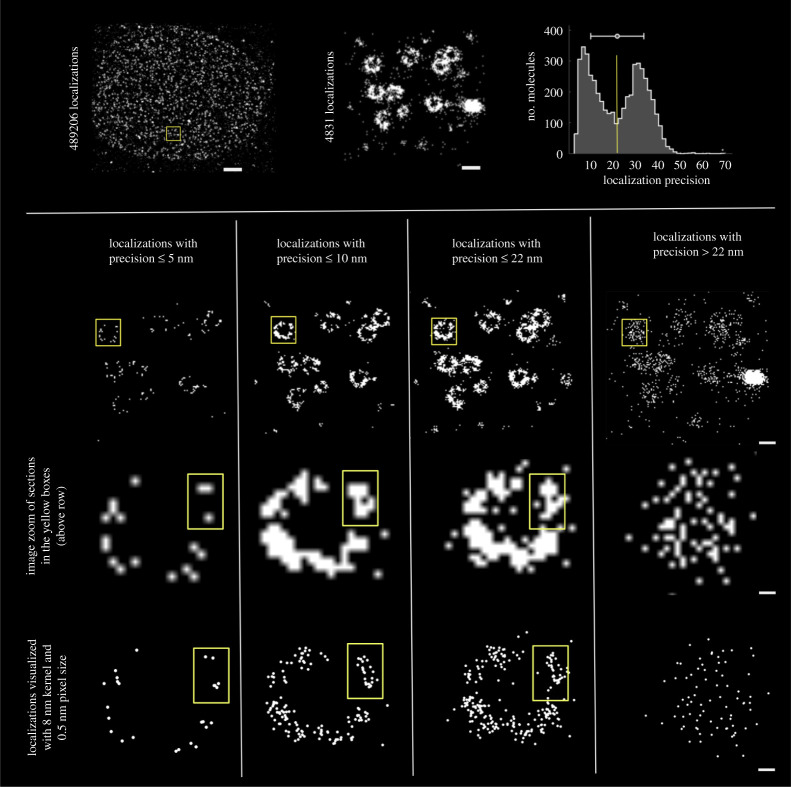
Filtering of localizations, visualization enhancement and molecular resolution: *(Top row)* (Left) Nuclear pore complexes (NPCs) data from Thevathasan *et al.* [23], accessed from www.ebi.ac.uk/biostudies/BioImages/studies/S-BIAD8. Scale bar, 1000 nm. (Middle) A zoomed-in excerpt (yellow box) with 4831 localizations for further downstream analysis in the panels below. Scale bar, 100 nm. (Right) The localization precision plot for the zoomed-in excerpt, notice the unexplained bimodal distribution. *(Bottom row, all columns)* The same nuclear pore section with different filtering of localizations at a manually chosen threshold (Scale bar, 100 nm). The zoomed-in excerpts of individual pores (yellow box) highlight the impact of visualization and image rendering (Scale bar, 10 nm). Note how the individual components of the eight subunits of NPCs which cannot be resolved with Gaussian blurring, can now be resolved with a manually chosen pixel size and Gaussian kernel (bottom row). The choice of pixel size, Gaussian kernels were done with *a priori* understanding of how the NPCs ‘should’ look like. The filtering threshold for localization precision was chosen to provide close to four copies per subunit. Often, in such cases, the missing copies are attributed to the limited efficiency of the labelling method. (Online version in colour.)

To highlight the role of localization filtering and *a priori* information to achieve molecular resolution, I use nuclear pore data from [23], available at www.ebi.ac.uk/biostudies/BioImages/studies/S-BIAD8. For downstream analysis at individual nuclear pore level, I zoomed-in at an image section with 4831 localizations (figure 4). For these localizations, the observed bimodal precision plot (centre at 22 nm) was used for further filtering of localizations. The same nuclear pore section with different filtering of localizations at a manually chosen threshold is shown in the bottom row, all columns (figure 4).

A single nuclear pore (yellow box) is further highlighted to show the impact of visualization and image rendering. Note how the individual components of the eight subunits of NPCs cannot be resolved with image zoom but can be resolved with a manually chosen pixel size (0.5 nm) and Gaussian kernel (8 nm). The choice of pixel size and Gaussian kernels were done with *a priori* understanding of what the NPCs ‘should’ look like. The filtering threshold (5 nm) was chosen to provide close to four copies per subunit. In this regard, we question the choice of pixel and kernel size used in Gwosch *et al.* [20]. For example, a pixel size of 0.5 nm and a large-width Gaussian kernel of 4 nm was used for figures 4*a* and 6*e* but a kernel of 2 nm and a pixel size of 0.2 nm for figure 4*a*, *f* in Gwosch *et al.* [20].

### (b) The filtered MINFLUX data

The MINFLUX filtering is done at various levels based on photon counts, targeted coordinate pattern to account for true emission and those considered as background events. For a fair comparison, both raw and filtered data should be presented to demonstrate that any such filtering is not biased by *a priori* structural information. For structures with prior information like NPCs, the manual filtering can lead to additional biases for live imaging, see figure 6*b*. For structures with little prior information, the reduction in the number of molecules would lead to under-sampled structure (figure 6*a*). Furthermore, it raises questions on the authenticity of the structures if an independent validation via electron microscopy or other super-resolution methods is not provided.

**Figure 5.**
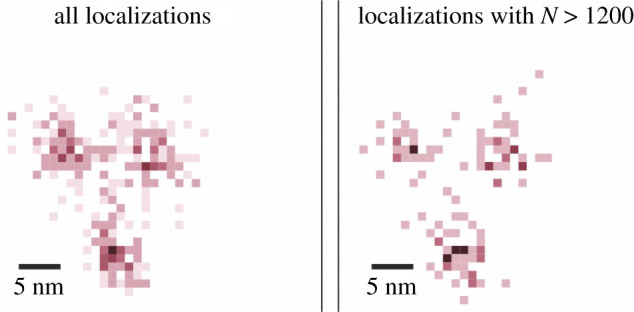
MINFLUX WYAKIWYG: what you already know is what you get. To demonstrate the difference between the experimental precision and the precision of the filtered data, I use p-MINFLUX data [26] provided by Florian Steiner. Masullo *et al.* [26] state that to achieve a certain precision (2 nm), a minimum number of photons (greater than 1200) are needed and is the primary reason for the choice of filtering threshold. For most biological samples, the underlying structure is almost always unknown, unlike here where the arrangement of DNA origami in 3-dots is known in advance. This can lead to subjective filtering thresholds for higher spatial resolution claims as long as 3-dots can still be distinguished. What if the arrangement of DNA origami was a T or Y shape? Would the same filtering with events greater than 1200 photons be still done? I recommend use of blind samples with no knowledge about the prior arrangement of DNA origami for proper resolution calibration. Note that the average precision of all the localizations would be much higher and this number has not been reported by the authors. The precision of all localizations is the true precision of the instrument and not the one calculated on the filtered data. (Online version in colour.)

Lastly, it is not clear if the molecular components of NPCs will still be visible in raw/unfiltered data as the increased density of signals will tend to overlap like in the two-colour images (figure 2). It is worth noting that subjective manual filtering with better localized molecules (as in figure S4 of Gwosch *et al.* [20]) leads to an increased resolution estimate (see figure 5 for a comparison).

**Figure 6.**
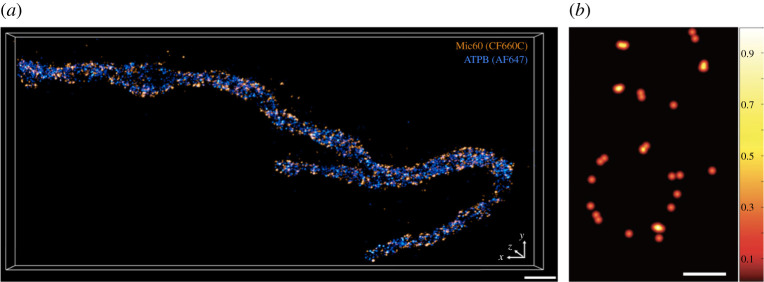
MINFLUX imaging of densely filled ring-like structures of Mic60 and Nup96: (*a*) Pape *et al.* [25] claim to observe ring-like arrangements of Mic60 molecules with a diameter of 40–50 nm. The ring-like arrangement is rather subjective here and is left for the readers to interpret themselves (see Movie http://movie-usa.glencoesoftware.com/video/10.1073/pnas.2009364117/video-6 for more insights). As no independent validation of these ring-like strictures via electron microscopy or other super-resolution methods is provided in the paper, the observed punctuated structures can, at best, be considered as a highly under-sampled ring. This raises questions on the detection efficiency of the method and its ability to image dense as well as continuous structures. Scale bar, 500 nm (*b*) Live MINFLUX imaging of nuclear pores expressing Nup96–mMaple in U2OS cell. Note the highly under-sampled configuration of nuclear pores as only the localizations with more than 1600 photons were considered. Image adapted from Gwosch *et al.* [20]. Scale bar, 50 nm. (Online version in colour.)

### (c) DNA origami and the need for blind samples

DNA origami is commonly used to measure the spatial resolution for different microscope setups. DNA origami has a pre-defined arrangement and blind samples with no knowledge on the prior arrangement are needed for proper calibration. This will make the photon count-based filtering more accountable as origami samples, unlike biological samples, have almost zero background (see figure 5). As signal-to-noise ratio is arguably the most important aspect in biological imaging, the effectively achieved resolution is heavily dependent on it and and can vary across sample regions. It is important to understand that unlike an implied single maximum resolution value, the effective resolution is variable, in particular due to the fluctuating contribution of light scatter and out-of-focus blur in different sample regions within the field of view.

Results obtained by imaging and analysing DNA origami will likely be a poor predictor of performance for real biological samples where problems of out-of-focus blur, non-specific background, light scattering and other sample aberrations exist. Thus, the resolution claimed based on DNA origami needs to be thoroughly investigated in this context and efforts made not to mislead the researchers interested in real biology.

In fact, a new quality metrics for microscopy techniques of the relative deviation between nominal and real life resolution in different biological situations needs to be introduced. After all, conventional fluorescence microscopy rarely achieves its theoretical limit. However, the relative deviation is much smaller than in MINFLUX.

### (d) The choice of biological sample for resolution claims

For the lateral spatial resolution, so far, MINFLUX imaging has been done on NPCs [20], microtubules [24], mitochondrial MICOS proteins [25] and post-synaptic protein PSD-95 [20]. These can be classified as samples having well-defined underlying structures (NPCs, microtubules) or with no inherent underlying structures (MICOS, PSD-95).

For the biological targets with no underlying structure, an independent validation with another super-resolution method or a relative comparison under identical imaging conditions and length scales is essential. For such studies, a minimum signal density and labelling efficiency should be considered as a minimum requirement.

For the biological targets with an underlying organization such as NPCs, the filtering of data can be misused to attain a certain resolution (figure 5). Furthermore, such filtering would lead to biases in labelling and detection efficiency.

## 4. Multi-colour, three-dimensional, live MINFLUX imaging and caveats when imaging continuous well-defined structures

MINFLUX was originally published in 2017 [10] with nanometer resolution claim on a synthetic structure (DNA origami) and particle tracking in bacterial samples. In Gwosch *et al.* [20], the authors, for the first time, claim nanometer resolution in biological samples, in three dimensions, in living cells and using multiple colour channels. In this section, I evaluate the method’s potential and the strength of the reported data.

### (a) The missing molecular components in multi-colour imaging

The individual proteins of the eight NPC subunits (roughly 40 nm apart) that can be observed in single-colour images, vanish in two-colour images (figure 2), putting into question the performance and resolution claim of MINFLUX under these imaging conditions. The molecularly resolved four copies of the subunits now appear as a ‘blob’ in two-colour images. This also includes the inner ring of the WGA displayed as a blob in the second colour channel. Note that both the inner and outer ring are clearly visible in Löschberger *et al.* [22] and Thevathasan *et al.* [23], respectively. In summary, for two colour imaging experiments, MINFLUX appears to offer an effective lateral resolution that is no better than localization-based super-resolution methods (see figure 2 for a detailed comparison). Can the molecular resolution still be claimed given that no ‘molecules’ are observed in a two-colour MINFLUX imaging experiment?

### (b) The rendered three-dimensional MINFLUX data

Regarding the three-dimensional imaging of well-defined structures like NPCs, the rendered MINFLUX data appears to be highly clustered, under-sampled and unevenly distributed (see figure 1, fifth row for a comparison with schematic of three-dimensional organization of NPCs). Several SMLM papers have put out a high-quality three-dimensional structure of NPCs and it is difficult to compare them with the rendered data from Gwosch *et al.* [20]. The under-sampled data again raises questions on detection efficiency of MINFLUX and its applicability for nuclear pore biology. It is also noted that no quantitative comparison of MINFLUX for Z resolution with SMLM is provided. For excellent three-dimensional nuclear pore data, please refer to figure 1 of Thevathasan *et al.* [23].

The two-colour three-dimensional images of the nuclear pores with WGA (CF680) and Nup96 (SNAP-Alexa Fluor 647) again are barely comparable with standard three-dimensional SMLM images. The ring distribution of WGA is unresolved and instead appears like a random distribution of points. The Nup96 octamer is also hardly visible, both laterally and axially (see figure 5 and Supplementary Video 3 of Gwosch *et al.* [20]).

### (c) MINFLUX imaging of continuous well-defined structures

MINFLUX has been used to probe the sub-mitochondrial localization of the core MICOS proteins [25]. The authors claim to observe ring-like arrangements with a diameter of 40 to 50 nm. Upon close examination, it appears that ring-like arrangement is rather subjective and the readers are encouraged to interpret it themselves, see figure 5*a* (this paper) or figure 3A and Movie S6 (http://movie-usa.glencoesoftware.com/video/10.1073/pnas.2009364117/video-6) of Pape *et al.* [25]. As no independent cross-validation of these ring-like strictures via electron microscopy or other super-resolution methods is provided, it raises concerns on the validity of the observed punctuated structures. These punctuations, at best, can be described as a highly under-sampled ring. At this point, it is not clear if this is due to the detection efficiency of MINFLUX and its limitation to image dense as well as continuous structures. This, in turn, highlights the need for sequential correlative super-resolution methods where new structural findings from one method can be independently validated under similar imaging conditions. Lastly, the claimed localization precision of 5 nm with a structural resolution of 40–50 nm brings in the debate of the difference between experimental ‘localization precision’ versus achieved practical ‘structural resolution’ and how these numbers should be reported.

*The microtubules:* Historically, FWHM of microtubules (MTs) has been the benchmark for resolution demonstration in STED microscopy. MTs are continuous and have a denser tubular organization (25 nm) when compared to the nuclear pores. Hence, any subjective filtering would lead to an under-sampled image. The hollow tube structure of MTs has been resolved with SMLM and DNA-PAINT setups. For well resolved three-dimensional cylinders of microtubules with SMLM, see Huang *et al.* [27], Li *et al.* [28]. On the other hand, MINFLUX fails to achieve the same level of structural resolution, see figure 3. Gerasimaite *et al.* [24] claim 15 nm for the inner diameter of microtubule, whose interpretation is left to the readers (standard estimate of inner diameter is 17 nm).

In summary, MINFLUX performance on continuous structures with a well-defined organization, such as MTs or those for which prior information is not available, remains to be proven. For MICOS, post-synaptic proteins, etc. which do not have an underlying structure, any resolution and shape can be claimed but is it valid? Thick samples where background is high will be another big challenge for MINFLUX.

### (d) Live MINFLUX imaging

MINFLUX imaging of nuclear pores (Nup96–mMaple) in living U2OS cells shows a highly under-sampled configuration with no data on cell viability or photo-bleaching (figure 5*b*). The observed uneven distribution of nuclear pores is possibly due to heavy filtering of localizations (greater than 1600 photons), see Prakash & Curd [29] for a detailed comparison.

Regarding excitation powers for live imaging, the authors used powers ∼20−60 μW with the peak intensities of the donut beam $∼10-50\;{\mathrm{kW}\;\mathrm{cm}}^{-2}$. For single-molecule techniques, the excitation power is $∼0.1\;{\mathrm{kW}\;\mathrm{cm}}^{-2}$ to switch the fluorophores between dark and bright state, ∼1000-fold less [8]. For structured illumination and confocal microscopes, especially the laser-free versions, the excitation powers are in tens of $mW/cm^{2}$ range or below. Thus, for live imaging MINFLUX is still off by a significant margin with respect to excitation powers when compared to single-molecule imaging, structured illumination or confocal microscopy.

## 5. Spatial resolution and localization precision

**Figure 7.**
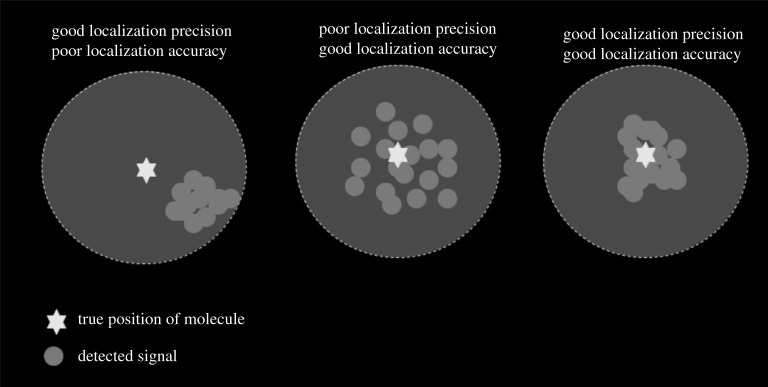
Localization precision of the fluorophore or the protein of interest: the readers of super-resolution microscopy papers should look out when localization precision is implied for spatial resolution. Resolution primarily depends on how densely a biological sample is labelled while localization precision implies how well these labels are detected. Image adapted from Prakash [30].

MINFLUX nanoscopy claims to provide a ‘resolution in the range of 1–3 nm for structures in fixed and living cells’. It is important to remember that 1–3 nm range here refers to localization precision, and more specifically of fluorophore and not that of the target protein (figure 7). As argued in this paper, MINFLUX is yet to resolve structures below 40 nm in biological samples. For synthetic structures like DNA origami, subjective event filtering can lead to unrealistically high precision numbers.

### (a) Precision is not resolution

For years, localization precision and spatial resolution have been interchangeably used by physicists given their inherent dependence on photons. Resolution, i.e. the ability to resolve close-by structures or target molecules, primarily depends on how densely a biological structure is labelled while localization precision implies how well these labels are detected, see figure 7 for more details.

For single-molecule studies, localization precision follows an inverse square-root relationship with the total number of detected photons, whereas MINFLUX has an inverse quadratic relationship with detected photons under ideal conditions with zero background. For real-life biological applications, MINFLUX is severely limited by the background photons as this compromises the positioning of the donut zero. In general, MINFLUX is likely to achieve better precision numbers than single-molecule imaging but so far, due to multiple reasons stated in this article, the spatial resolution of the method has been much poorer than SMLM methods (figure 1).

### (b) Resolution quantification

Gwosch *et al.* [20] applied three different criteria for resolution quantification. The first approach was to calculate the standard deviation for >5 localizations/fluorophore, where each localization had ∼2000 photons. The events with high photon counts would automatically result in higher precision numbers. Note how Gwosch *et al.* [20] begin with resolution quantification but quote the precision of filtered data, skipping the average precision of all localizations. This leads to confusing localization precision of a fluorophore with the spatial resolution of the method.

The second resolution assessment is made by subtracting the mean localized position from all localizations. This process is similar to the first approach and will be biased by the filtering of the data. The third approach is based on Fourier ring correlation (FRC) and it is not clear whether filtered or raw data was used for FRC analysis. FRC measures correlation between subset of localizations and will again be affected by filtering of the data.

Prakash & Curd [29] estimated localization precision ($\sigma_{xy}$) were 0.98±0.02 nm, 3.20±0.05 nm and 3.31±0.08 nm for the two-dimensional, three-dimensional 1-colour and three-dimensional 2-colour MINFLUX datasets [20], respectively. FWHM≈2.355σ, so $\sigma_{xy}$ of 1–3 nm implies FWHM of 2.4–7.1 nm for a single molecule. So, one would not expect to clearly resolve molecules closer than this.

### (c) 1-nm resolution and 3-nm linker

A resolution cannot be better than the localization precision of the detected fluorophore. As a general rule, a more conservative upper limit for localization precision or full-width at half-maximum should be used to account for the linkage errors. From a biochemistry point of view, how reasonable to have a resolution in the range of 1–3 nm when the SNAP-tag itself is about 3–3.5 nm in size? Given the resulting uncertainty of relative position of the dye molecule, the stated nanometer resolution (or the localization precision for that matter) can only be claimed for the fluorophore, but not the biological target. In this context, I urge the readers to take careful note when authors state the precision of the fluorophore and that of the target protein.

### (d) Is it all about the principle and not practice?

Generally speaking, the resolution is the fundamental ability to determine a structure. From a physicist perspective, it is satisfying that MINFLUX can achieve 1-nm localization precision under ideal conditions (based on photon statistics), whether it materializes in resolving real life biology or not. From a biologist perspective, for a 1-nm resolution claim, MINFLUX is off by a factor of 5 for synthetic samples like DNA origami and a factor of 40 for biological samples like NPCs.

For a true 10 nm structural resolution, a general expectation would be to resolve a well-established 10 nm bead-on-string structure of nucleosomes or a turn of DNA helix which is about 3.4 nm (for 1-nm claim). From Nyquist viewpoint to achieve 10 nm structural resolution, one would need a dye every 4 nm, which for many biological structures is hard to achieve due to steric hindrance or low copy number/binding sites. Alternatively, for structural biology, with true 1-nm resolution claim, I wonder if one can compare MINFLUX with X-ray crystallography, which has a broad range of resolution (anything between 1.5 and 10 Å) depending on the crystal quality.

## 6. Summary and the lessons from the past

### (a) Towards standard biological reference structures

The spatial resolution in light microscopy has become something of a numbers game where scientists cite an arbitrary FWHM or an FRC resolution or filter localizations to achieve any desired resolution. Given the large number of potential biological reference structures in the range of 1–100 nm size scale, for example, DNA (2 nm), nucleosomes (10 nm), microtubules (20 nm), NPCs (40 nm), synaptonemal complexes (60–150 nm), I recommend using these well-defined structures to support spatial resolution claims. For DNA origami researchers, I encourage usage of blind samples where the origami arrangement or the distances are not known in advance. This will make the photon count-based filtering more accountable.

### (b) Potential research areas for MINFLUX

Gwosch *et al.* [20] and Balzarotti *et al.* [10] have made efforts to push the localization precision to single nm range which works well on ideal synthetic samples, but major compromises need to be made when applying these techniques to real-life biology. MINFLUX has value for a certain specific applications (e.g. single-molecule tracking, material science i.e. nitrogen-vacancy centres), but in its current state, it should not be considered as the next-generation versatile imaging platform. Technical issues with detection, limitation when imaging for well-defined, continuous and dense structures, the use of well-characterized single-molecule dyes still limits its broader applicability.

### (c) Reproduction crisis

MINFLUX papers have shown several loop-holes in current scientific reporting and standards. A clear distinction needs to be made about experimental precision, spatial resolution, cross-validation of new structures by independent methods, extent of sample variability versus measurement uncertainty, hardware adaptability, image quality/standards (for example, acceptable measures for image rendering), availability of raw data/codes, reproducibility of the analysis pipeline, automated versus manual components of data analysis (like filtering) and proper procedures to report them [31].

From a historical perspective, 4Pi, STED and now MINFLUX have been closed systems with little focus on making the hardware adaptable or raw data/codes available. Several units of highly priced 4Pi microscopes were sold around 2000s as the next generation microscope, however, not many biological findings resulted. This was followed with the launch of STED microscopes with very few novel biological results.

Science is about reproducible measurements. Improvement in precision leads to refined measurements. MINFLUX improves the precision with which fluorophores can be localized but by not making the hardware adaptable, raw data and codes available, it is hindering reproducibility, open science efforts and overall progress of the field.

### (d) Important conceptual advancement versus current technical limitations

One of the important aspects of science is to promote cutting-edge ideas even if they offer few gains in the short run. MINFLUX research provides an important conceptual advancement and leads to overall progress in the field. The aim of this article is not to discourage MINFLUX research but to highlight that the technology is still underdeveloped and needs further validation from other independent labs before it is made commercially available, especially considering the high price tag of the commercial system.

As research grants mostly come from tight public funds and are a zero-sum game, I hope this critique provides the scientists, microscope users, developers and core facility managers with an alternative viewpoint when deciding about an investment in the next ‘state-of-the-art’ microscope instrument.

## Data accessibility

All data published elsewhere has been cited and the relevant journal permissions were taken.
